# Genome-wide characterization of PEBP gene family in *Perilla frutescens* and *PfFT1* promotes flowering time in *Arabidopsis thaliana*

**DOI:** 10.3389/fpls.2022.1026696

**Published:** 2022-11-18

**Authors:** Huaxiang Xu, Xi Guo, Youjin Hao, Geng Lu, Dan Li, Junxing Lu, Tao Zhang

**Affiliations:** Chongqing Key Laboratory of Molecular Biology of Plant Environmental Adaptations, College of Life Sciences, Chongqing Normal University, Chongqing, China

**Keywords:** Perilla frutescens, flowering locus T, PfFT1, PEBP family, flowering time

## Abstract

Phosphatidylethanolamine-binding proteins (PEBP) family plays important roles in regulating plant flowering time and morphogenesis. However, geneme-wide identification and functional analysis of *PEBP* genes in the rigorous short-day plant *Perilla frutescens* (*PfPEBP*) have not been studied. In this study, 10 PfPEBP were identified and divided into three subfamilies based on their phylogenetic relationships: FT-like, TFL1-like and MFT-like. Gene structure analysis showed that all *PfPEBP* genes contain 4 exons and 3 introns. Motifs DPDxP and GIHR essential for anion-binding activity are highly conserved in PfPEBP. A large number of light-responsive elements were detected in promoter regions of *PfPEBP*. Gene expression of *PfFT1* exhibited a diurnal rhythm. It was highly expressed in leaves under the short-day photoperiod, but higher in flowers and seeds under the long-day photoperiod. Overexpression of *PfFT1* in *Arabidopsis thaliana* not only promoted early flowering of Col-0 or Ler, but also rescued the late flowering phenotype of *ft-1* mutant. We concluded that *PfFT1* promotes early flowering by regulating the expression of flowering-related genes *AtAP1*, *AtLFY*, *AtFUL* and *AtSOC1*. In conclusion, our results provided valuable information for elucidating the functions of *PfPEBP* in *P*. *frutescens* and shed light on the promoting effect of *PfFT1* on flowering.

## Introduction

Phosphatidylethanolamine-binding proteins (PEBP) are widely-distributed in animals, plants and microorganisms. They contain a conserved PEBP domain and play important roles in regulating plant flowering, seed development and germination (Yu et al., 2019; Cheng et al., 2021). Currently, the PEBP gene family is divided into three subfamilies: MOTHER OF FT AND TFL1-like (MFT-like), FLOWERING LOCUS T-like (FT-like), and TERMINAL FLOWERING 1-like (TFL1-like). MFT-like was thought to be the common ancestor of FT-like and TFL1-like, which emerged during the evolution of seed plants (Hedman et al., 2009). *MFT* genes were highly expressed in seeds and involved in seed development and germination by regulating abscisic acid (ABA) and gibberellin (GA) signaling pathways (Xi et al., 2010). FT-like and TFL1-like subfamily members were involved in the regulation of flowering time and morphogenesis. The TFL1-like subfamily includes *TFL1*, *CENTRORADIALIS* (*CEN*) and *BROTHER OF FT AND TFL1* (*BFT*). *TFL1* could inhibit the formation of flower primordia, thus delaying flowering. *TFL1* inhibited flowering by binding to the bZIP type transcription factor *FLOWERING LOCUS D* (*FD*) (Kaneko-Suzuki et al., 2018; Freytes et al., 2021; Zuo et al., 2021). Introduction of apple (*Malus* × *domestica*) *MdTFL1* or *MdTFL2* genes into *Arabidopsis* could significantly delay flowering and increase the number of rosette leaves and plant height (Zuo et al., 2021). Overexpression of *PgTFL1* and *PgCENa* of pomegranate (*Punica granatum*) were able to suppress the flowering defect of *Arabidopsis tfl1-14* mutant (Patil et al., 2018). In rice (*Oryza sativa*), OsCEN and Hd3a (Heading date 3a, an ortholog of FT protein) competitively bind to OsFD and regulate flowering (Kaneko-Suzuki et al., 2018).

The FT-like subfamily includes two members, *FT* and *TWIN SISTER OF FT* (*TSF*). The FT-FD complex can activate the expression of flowering-related genes, such as *FRUITFULL* (*FUL*), *LEAFY* (*LFY*), *APETALA1* (*AP1*) and *SUPPRESSOR OF OVEREXPRESSION OF CO1* (*SOC1*) (Abe et al., 2005; Wigge et al., 2005; Lee and Lee, 2010). In the LD plant *Arabidopsis*, overexpression of *AtFT* could significantly rescue the delayed flowering of *ft-1* mutant (Ruiz-García et al., 1997; Kardailsky et al., 1999). In the short-day (SD) plant rice, Hd3a-OsFD1 complex could activate the expression of *OsMADS15* (a homologue of *AP1*), and promoted the flowering (Taoka et al., 2011). At present, homologous genes of *FT* have been identified in several higher plant species, including pear (*Pyrus communis*) (Zhang M. et al., 2021), cotton (*Gossypium hirsutum*) (Wang et al., 2019), sugarcane (*Saccharum* spp.) (Venail et al., 2022), rice (Kaneko-Suzuki et al., 2018), and *Arabidopsis* (Wigge et al., 2005). Growing evidences showed that *FT* genes play crucial roles in plant flowering. In tobacco (*Nicotiana tabacum*), *NtFT1*, *NtFT2* and *NtFT3* inhibited flowering, while *NtFT4* could promote flowering (Harig et al., 2012), which was caused the mutations of three key amino acids in the PEBP domain. Functional analysis revealed that the sugarcane *ScFT3* gene could rescue the late flowering phenotype of the *Arabidopsis ft-10* mutant, but *ScFT5* could not (Venail et al., 2022). In addition, *FT* genes can also regulate developmental processes other than the floral transition. In onion (*Allium cepa*), *AcFT1*, *AcFT5* and *AcFT6* regulate bulb formation under long-day (LD) conditions (Rashid et al., 2019). Two *FT*-like paralogues (*StSP3D* and *StSP6A*) could respond to different environmental cues and regulate flowering and tubers formation in potato (Navarro et al., 2011).

*Perilla* (*Perilla frutescens*) seed oil is rich in unsaturated fatty acids. The content of α- linolenic acid is up to 60%, which has important application in medicine and food industry (Liao et al., 2018). Additionally, *Perilla* is a rigorous SD plant (King and Zeevaart, 1973), making it an ideal species for exploring the effect of photoperiod on flowering. The PEBP gene family of *P. frutescens* (PfPEBP) has not been identified and their functions are still unknown. The recently released *P. frutescens* genome provides important data for solving above questions (Zhang Y. et al., 2021). In this study, the members of *PfPEBP* genes were identified and systematically analyzed at the genome-wide level, including phylogenetic relationship, gene structure and chromosomal distribution. The effect of photoperiod on *PfFT1* gene expression, as well as its diurnal rhythm and tissue specificity, was analyzed. Its role in promoting flowering was further explored by transforming *PfFT1* into *Arabidopsis*. In conclusion, our findings will help to understand the molecular mechanism of the regulatory roles of *PfFT1* in flowering control of *Perilla*.

## Materials and methods

### Identification of PEBP gene family members in *P. frutescens*

The annotated genome data of *P. frutescens* was obtained from the GenBank (https://www.ncbi.nlm.nih.gov/genbank/). Amino acid sequences of *Arabidopsis* and rice PEBPs were retrieved from the TAIR (http://www.arabidopsis.org/) and RGAP (http://rice.uga.edu/) databases, respectively. To identify PfPEBP, the Hidden Markov Model (HMM) profile of the PBP domain (PF01161) was obtained from the Pfam database and used as the query. In addition, amino acid sequences encoded by the genome of *P. frutescens* were also searched using HMMER v3.3.2 (E-value ≤ 1.0 × e^−5^). BLASP was performed against *P. frutescens* genome using *Arabidopsis* PEBPs as queries (E-value ≤ 1.0 × e^−5^). Finally, PfPEBP candidates were submitted to CD-Search (https://www.ncbi.nlm.nih.gov/Structure/cdd/wrpsb.cgi), SMART (http://smart.embl-heidelberg.de/) and Pfam (http://pfam.xfam.org/search) for conserved domain analysis.

### Sequence characterization

The basic physicochemical properties of PfPEBP were analyzed using the ProtParam tool in Expasy (https://www.expasy.org/), including putative molecular weight (MW), isoelectric point (*p*I), and grand average of hydropathicity (GRAVY). Subcellular localizations were predicted using the Plant-mPLoc prediction tool (http://www.csbio.sjtu.edu.cn/bioinf/plant-multi/). The chromosomal mapping was performed based on genome annotation information and visualized with the TBtools (Chen et al., 2020). Gene structure was analyzed using the GSDS 2.0 server (http://gsds.gao-lab.org/). Finally, the conserved motifs were predicted using the MEME Suite (https://meme-suite.org/meme/tools/meme) with the following parameters: optimum width 10–50 amino acids, any number of repetitions of a motif, and maximum number of motifs set at 5.

### Multiple sequence alignments and phylogenetic analysis

Amino acid sequences of PfPEBP were aligned using the Clustal Omega and visualized using the Jalview. Phylogenetic tree of 25 PEBP proteins from *Perilla*, *Arabidopsis* and rice were constructed using the Neighbor-Joining (N-J) method (1000 bootstrap replicates) in MEGA X based on JTT+G substitution matrix. The phylogenetic tree was visualized with the iTOL tool (https://itol.embl.de).

### *Cis*-acting elements analysis

To understand the *cis*-acting elements in the promoter region of *PfPEBP*, a 2000 bp upstream fragment of the initiation codon (ATG) was retrieved and predicted by the PlantCARE (http://bioinformatics.psb.ugent.be/webtools/plantcare/html/). In addition, the *cis*-acting elements of rice *OsFTL1*, *OsFTL2*, *OsFTL3*, and *Arabidopsis AtFT* were also predicted and visualized using the TBtools.

### Prediction of protein-protein interaction (PPI) network

The homologous proteins of PfFTs and PfTFL1s in *Arabidopsis* were determined by BLASTP. Subsequently, prediction of PPI networks with PfFTs and PfTFL1s as hub proteins were performed using STRING v11.5 (https://string-db.org/) (Szklarczyk et al., 2019).

### Plant materials and treatments

Seeds of *Perilla* and *Arabidopsis* [Col-0 (Columbia ecotype), Ler (Landsberg erecta ecotype) and *ft-1* mutant (Ler ecotype)] were kept in the Key Laboratory of Oil Peony Germplasm Innovation and Utilization of Chongqing Normal University. *Perilla* seeds were sown in plastic pots with nutrient soil and coarse soil (1:2, v:v) and grown in a incubator: 24°C/20°C (Light/Dark), 16 h/8 h (LD: Light/Dark) or 8 h/16 h (SD: Light/Dark). The cultivation and growth conditions of *Arabidopsis* (Col-0, Ler and *ft-1*) were performed as described by Xu et al. (Xu et al., 2022).

### Photoperiod effects on expression of *PfFT1* gene in different tissues

When grown to 6 true leaves, leaves were collected every 4 h for 48 h and used for diurnal rhythmicity analysis of *PfFT1* expression. To analyze the tissue specific expression patterns of *PfFT1* under different photoperiods, the roots, stems, leaves, flowers at flowering stage were collected at 56/141 days after sown (DAS), respectively. Mature seeds were collected at 86 DAS (SD condition), 171 DAS (LD condition). All samples were snap-frozen in liquid nitrogen and stored at −80°C until use.

To investigate the expression profiles of *PfFT1* in different tissues and photoperiodic conditions, quantitative real-time polymerase chain reaction (RT-qPCR) was performed. Total RNA was extracted using the RNAprep pure Plant Kit (Tiangen, Beijing) and reverse transcribed into cDNA using the PrimeScript RT Reagent Kit with gDNA Eraser (Takara, Japan). The RT-qPCR reaction system (20µL) contains 10 ng cDNA, 10 µL SYBR Green Supermix (BioRad, USA), 1 µL primer pairs (10 µM/each), and ddH_2_O. The reaction conditions were 95 °C for 2 min, followed by 40 cycles at 95 °C for 10 s, 60 °C for 20 s and 72 °C for 20 s. *Perilla PfActin7* was used as the internal reference gene. Three biological and three technical replicates (n=3×3) were performed for each sample. Primers used in this study were listed in **Supplementary Table S1**. The relative expression level of *PfFT1* was calculated using the 2^−ΔΔCT^ method (Livak and Schmittgen, 2001).

### Gene cloning, plant transformation, and transgenic lines screening

The full-length coding sequence of *PfFT1* was amplified using the leaf cDNA as the template. The PCR reaction system contains 1 µL cDNA, 10 µL Premix Taq enzyme (TAKARA, Japan), 1 µL primer pairs (10 µM/each), and 8 µL ddH_2_O. The reaction conditions were 94 °C for 5 min, followed by 30 cycles at 94°C for 30 s, 57°C for 30 s and 72°C for 60 s, and a final extension at 72 °C for 10  min. All PCR products were detected by 1% agarose gel electrophoresis, and purified using the SanPrep Column DNA Gel Extraction Kit (Sangon, Shanghai). The recombinant vector pCAMBIA1303-*PfFT1* was constructed using the In-Fusion HD Cloning Kit (Takara, Japan) following the manufacturer’s instructions, and then transformed into *Agrobacterium tumefdciens* GV3101 (WEIDI, China) by the freeze-thaw method. *Arabidopsis* Col-0, Ler and *ft-1* were transformed using the floral dip method (Clough and Bent, 1998). The harvested plant seeds were planted in 1/2 MS medium containing 20 mg/L hygromycin B. Plants that can grow 4 or more true leaves were considered as positive seedlings and transplanted to soil. Leaf genomic DNA was extracted and amplified against the T-DNA region of the pCAMBIA1303 vector to detect the target gene *PfFT1*. The transgenic *Arabidopsis* was then stained using the GUS stain Kit (Coolaber, China). Plants validated by the above two steps were regarded as the positive T_0_ generation. Following the same protocol, the T_3_ generation without trait segregation was considered as a homozygous transgenic line and used for further studies.

### Measurement of flowering time and expression level of flowering-related genes

To analyze the effect of *PfFT1* on flowering, wild-type Col-0, transgenic Col-0 (TC); *ft-1* mutant, complemented mutant (CM); Ler, and transgenic Ler (TL) plants were grown in an artificial climate chamber under the LD condition (22°C/20°C, Light/Dark). At least 10 plants from each line were selected to record the bolting and flowering time, number of rosette leaves at flowering.

Total RNA was extracted from leaves of 20-day-old *Arabidopsis* (transgenic or non-transgenic line) grown under the LD conditions, and reverse transcribed into cDNA and used for gene expression analysis of *PfFT1*, *AtFT*, *AtAP1*, *AtFUL*, *AtSOC1* and *AtLFY*. The RT-qPCR reaction system (20µL) consisted 10 ng cDNA, 10 µL SYBR Green Supermix, 1 µL primer pairs (10 µM/each), and ddH_2_O. The reaction condition was 95 °C for 1 min, followed by 40 cycles at 95 °C for 10 s, 60 °C for 30 s and 72 °C for 20 s. *Arabidopsis AtActin8* was used as the internal reference gene. Three biological and three technical replicates (n=3×3) were performed for each sample. Primers were listed in **Supplementary Table S1**. The relative gene expression levels were calculated using the 2^−ΔΔCT^ method.

### Statistical analysis

All data were analyzed by Student’s *t*-test using IBM SPSS v26.0. Significant difference was considered when *P* < 0.05. Graphs were plotted using GraphPad Prism v8.0.

## Results

### Identification and characterization of PEBP family genes in *P. frutescens*

A total of 10 *PfPEBP* genes were identified and named according to the similarity with the homologous genes in *Arabidopsis*, including 2 *PfFT*, 4 *PfTFL1*, 2 *PfCEN* and 2 *PfMFT* (**Supplementary Table S2**). The corresponding protein length ranges from 149 (PfCEN2) to 176 (PfMFT1) amino acids, the molecular weight of PfPEBP ranges from 17.0 to 19.9 kDa, and the *p*I ranges from 7.74 to 9.51. All GRAVY values were less than 0, indicating that all PfPEBP are hydrophobic. Subcellular localization prediction showed that PfFTs, PfTFL1s and PfCENs, and PfMFTs were located in the nucleus, cytoplasm, cytoplasm-nucleus, respectively (**Supplementary Table S2**).

**Figure 1:**
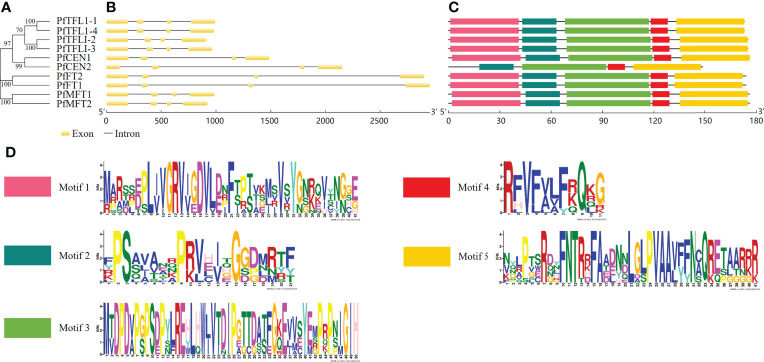
Gene structures and motifs of *PEBP* genes in *P. frutescens*. **(A)** The phylogenetic tree was constructed based on amino acid sequences of 10 PfPEBP. **(B)** Gene structures of *PfPEBP* genes was constructed using the GSDS 2.0 tools. Yellow boxes indicate exons and black lines represent introns. **(C)** Motif composition of PfPEBP. The conserved motif numbers in the MEME prediction was set to 5. **(D)** Sequence logos of five conserved motifs. The overall height of each stack indicates the sequence conservation at that position.

To understand gene structural diversities of *PfPEBP* genes, chromosomal location, number of exons and introns, and distribution of conserved domains were investigated. Chromosome mapping showed that 10 *PfPEBP* genes were distributed on Chr02, Chr05, Chr11, Chr14, Chr15, Chr17 and Chr18 (**Figure S1**). The phylogenetic relationships of the PfPEBP family was shown in **Figure 1A**. Each *PfPEBP* gene was composed of 4 exons and 3 introns (**Figure 1B**). Motifs 2-5 were conserved in all PfPEBP (**Figure 1C**). Only PfCEN2 lacks motif 1. Each motif contained 11-50 amino acids (**Figure 1D**). Members in the same subfamily shared a similar motif pattern, suggesting that they might have similar functions. Comparison analysis revealed that the conserved PEBP domain contained complete motifs 2-5, indicating that they are crucial for their functions, and also explaining the conserved domain patterns of PfPEBP members.

### Amino acid alignment and conserved domain analysis

**Figure 2:**
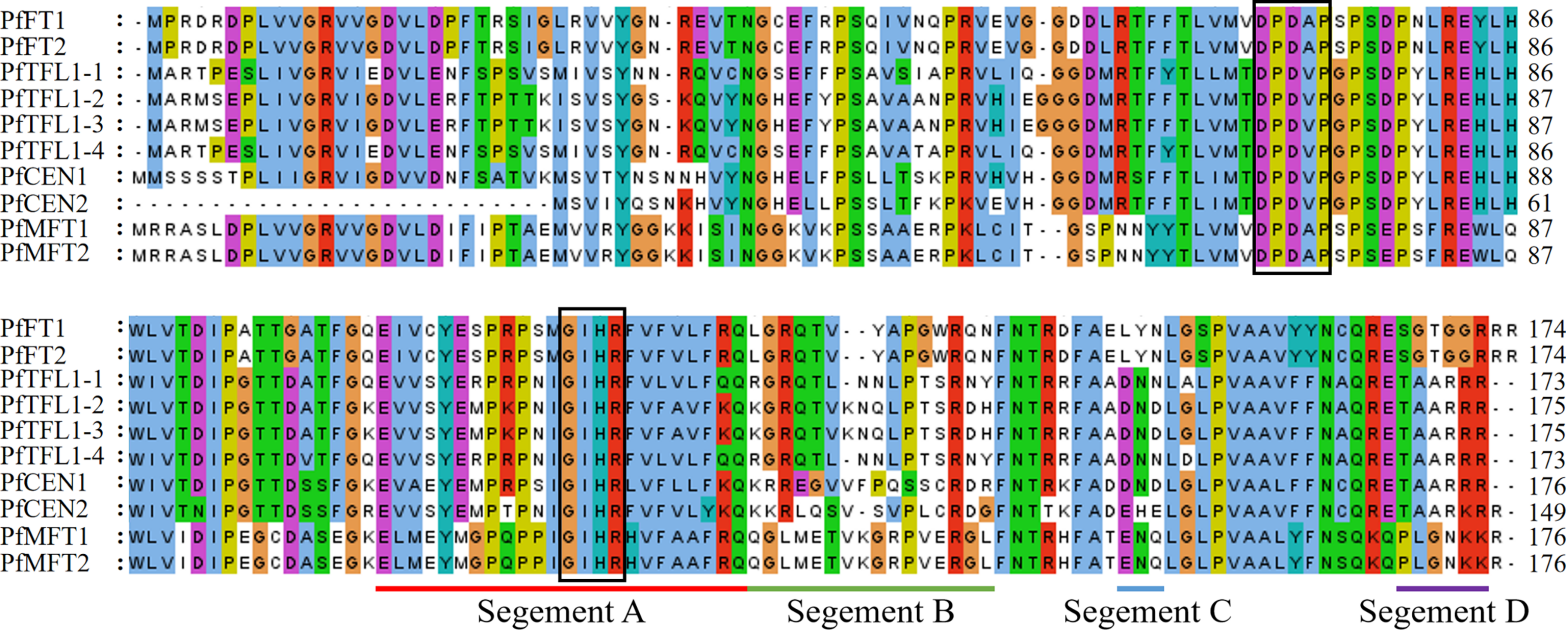
Multiple sequence alignment of PEBP in *P. frutescens*. Conserved motif DPDxP and GIHR was marked with black frame, respectively. Segment A–D was underlined with different colors.

Amino acid sequence alignment showed that the identities between PfFTs and PfTFL1s were more than 55% (**Figure 2**). All PfPEBP have conserved motif DPDxP and GxHR, which are critical for anion-binding. Particularly, motif GxHR has a preference for Ile residues. The conserved motif LYN in PfFTs was localized in segment C, which was essential for the enzymatic activity. Fragment D of PfFTs contains the motif SGTGGR, while that of PfTFL1s were replaced by the motif TAARRR. Further analysis showed that all PfFTs had the key amino acid residues Tyr^84^, Gln^139^, but they were replaced by His^85^, Asp^142^ or Asn^142^ in PfTFL1s.

### Phylogenetic analysis

**Figure 3:**
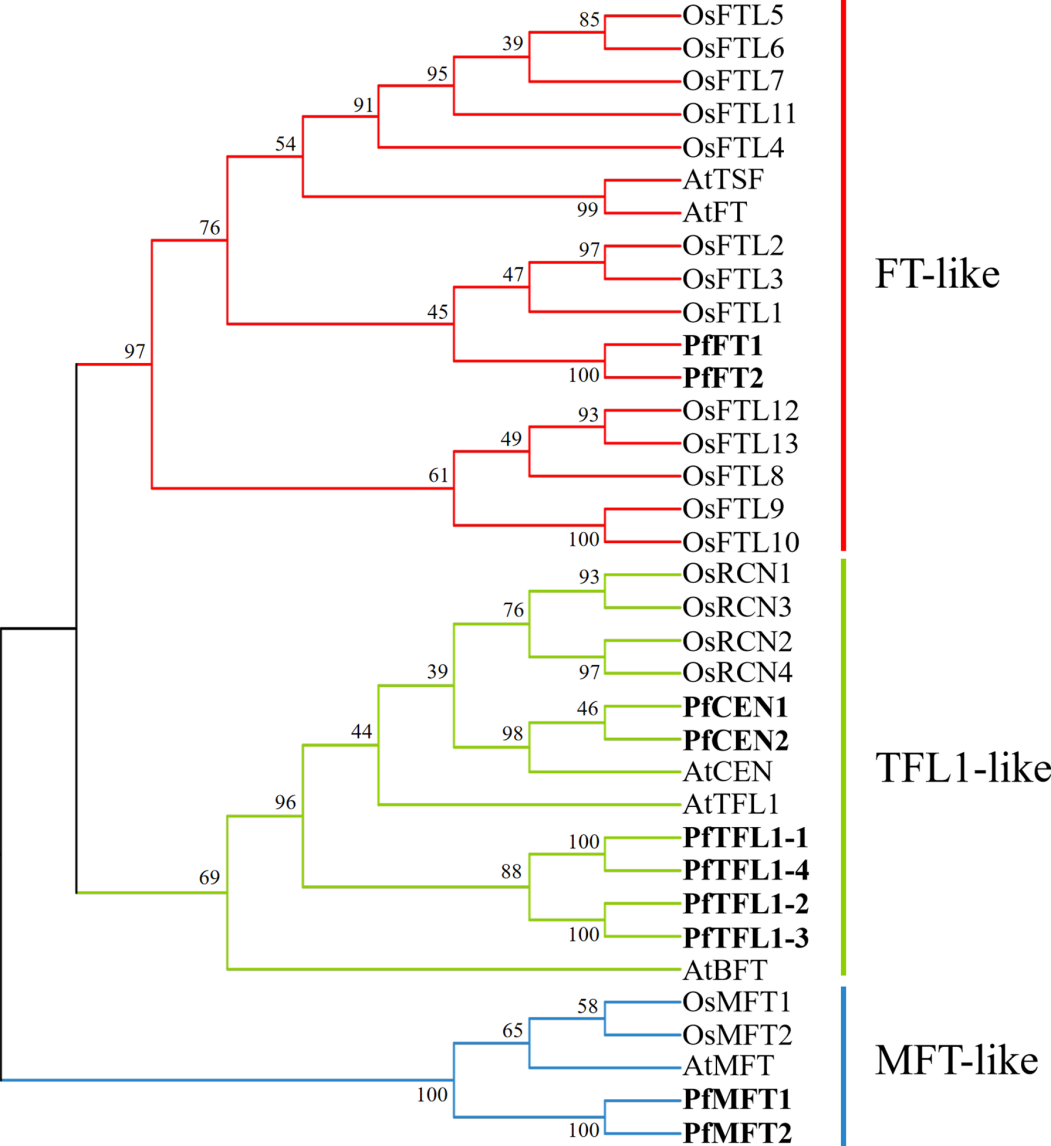
Phylogenetic relationship of PEBP in *P. frutescens*, *A. thaliana*, and *O. sativa*. The phylogenetic tree was constructed using the Neighbor-Joining method in MEGAX with 1000 bootstrap replicates. FT-like, TFL1-like, and MFT-like subfamilies were marked by red, green, and blue, respectively. PfPEBP were shown in bold font. GenBank accession number of each sequence was listed in **Supplementary Table S2** and **S3**.

To explore the evolutionary relationship of the PfPEBP family, a phylogenetic tree of PEBP from *P. frutescens*, *A. thaliana*, and *O. sativa* was constructed. The results showed that 35 PEBP proteins were clustered into three subfamilies: FT-like, TFL1-like and MFT-like (**Figure 3**). The FT-like subfamily has 18 members, including AtFT, OsFTLs, and PfFTs. The TFL1-like subfamily has 12 members, including AtCEN, AtTFL1, AtBFT, OsCENs, PfCENs and PfTFL1s. The MFT-like subfamily has five members, including AtMFT, OsMFTs, and PfMFTs.

### Analysis of *cis*-acting regulatory elements

**Figure 4:**
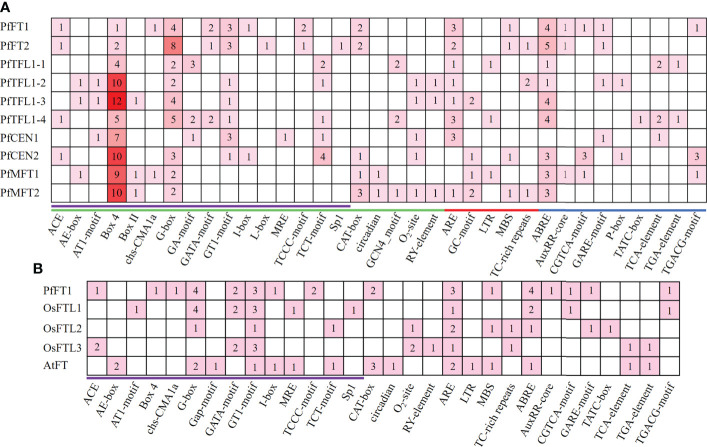
*Cis*-regulatory elements analysis. **(A)** *Cis*-regulatory elements analysis was performed in a 2000 bp promoter region of *PfPEBP* genes. Green, red, and blue lines indicate growth and development, biotic/abiotic stress responses, and hormone responsive element, respectively. Light-response elements were marked in purple. The numbers and colors represent the number of specific *cis*-acting elements in a gene. The larger the number, the darker the color, and the smaller the number, the lighter the color. **(B)** *Cis*-regulatory elements in the promoter region of *PfFT1*, *OsFTL1*–*3*, *AtFT*. Light-response elements were marked in purple.

To further understand the regulatory patterns of *PfPEBP* genes, the *cis*-regulatory elements within a fragment 2000 bp upstream of the start codon (ATG) of each gene were analyzed. Based on their functions, they were divided into three categories: growth and development, hormone responses, and biotic/abiotic stress responses (**Figure 4A** and **Supplementary Table S4).** Light-responsive elements, such as Box4 and G-Box were present in the promoter of all *PfPEBP* genes, suggesting that they are light signal reponse regulators. Circadian-responsive element (circadian) only occurred the promoter of *PfMFT* genes, whereas the anaerobic-inducible element (GC-motif) was only present in the promoter of *PfTFL1* and *PfMFT* genes. Further analysis found that the distribution of hormone response elements was quite different. The auxin responsive element (AuxRR-core) was only found in *PfFT* and *PfMFT* genes. These findings revealed that the *PfPEBP* genes could respond light, hormones and stresses.

Comparion analysis revealed that *Arabidopsis* (LD plant) has specific light-response elements, such as AE-Box and Gap-motif, while *Perilla* and rice (SD plant) have specific light-response elements, such as ACE, AT1-motif, Box-4, Chs-CMA1a, GATA-motif, TCCC-motif, and sp1 (**Figure 4B** and **Supplementary Table S5**). Differences in *cis*-acting elements in promoter regions of *PEBP* genes in LD and SD plants might lead to their different responses to photoperiod.

### PPI network

**Figure 5:**
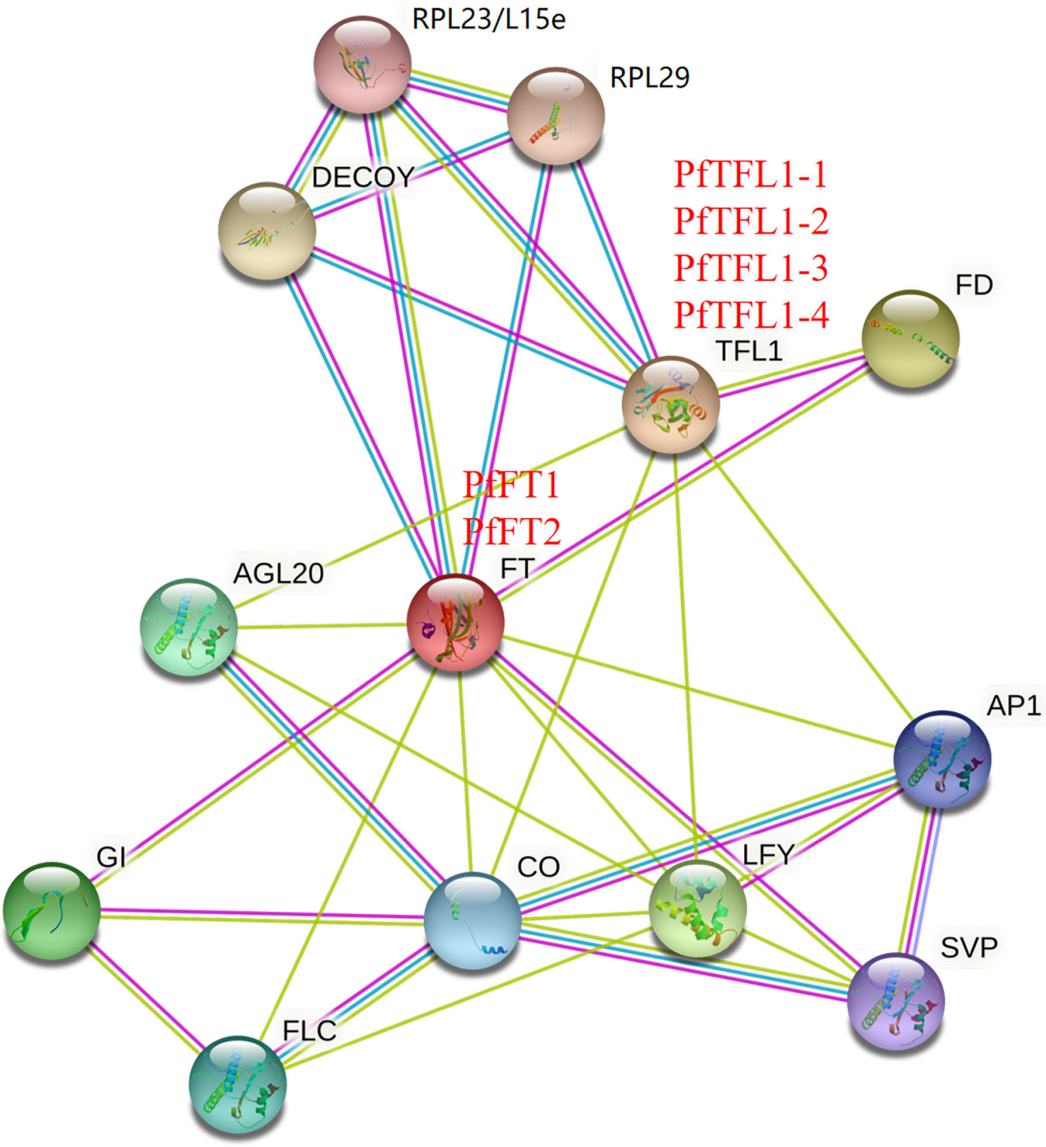
Protein-protein interaction network. The PPI network was predicted based on the homologous proteins in *Arabidopsis*. RPL23/L15e, Ribosomal protein L23/L15e family protein; RPL29, Ribosomal protein L29 family protein; DECOY, DECOY; FD, FLOWERING LOCUS D; TFL1, TERMINAL FLOWER 1; FT, FLOWERING LOCUS T; AGL20, AGAMOUS-LIKE 20; AP1, APETALA1; GI, GIGANTEA; CO, CONSTANS; LFY, LEAFY; SVP, SHORT VEGETATIVE PHASE; FLC, FLOWERING LOCUS C.

Comparison of amino acid sequences is helpful to understand functional similarities, amino acid sequence identities between PfFTs and AtFT was 78.16%, and that of PfTFL1s and AtTFL1 was 68.60-72.83% (**Figure S2** and **Supplementary Table S6**). High sequence identity and conservation of key motifs provided an important basis for PPI network prediction, which provides a more intuitive understanding of their functions. STRING analysis showed that both PfFTs and PfTFL1s potentially interact with FD, AP1, LFY, CO, AGL20, DECOY, RPL23/L15e, and RPL29. In addition, PfFTs also interacted with FLC, GI, and SVP (**Figure 5**). This result suggested that PfFTs and PfTFL1s may have different functions in regulating flowering through similar pathways.

### Photoperiod effects on expression patterns of *PfFT1* genes in different tissues

**Figure 6:**
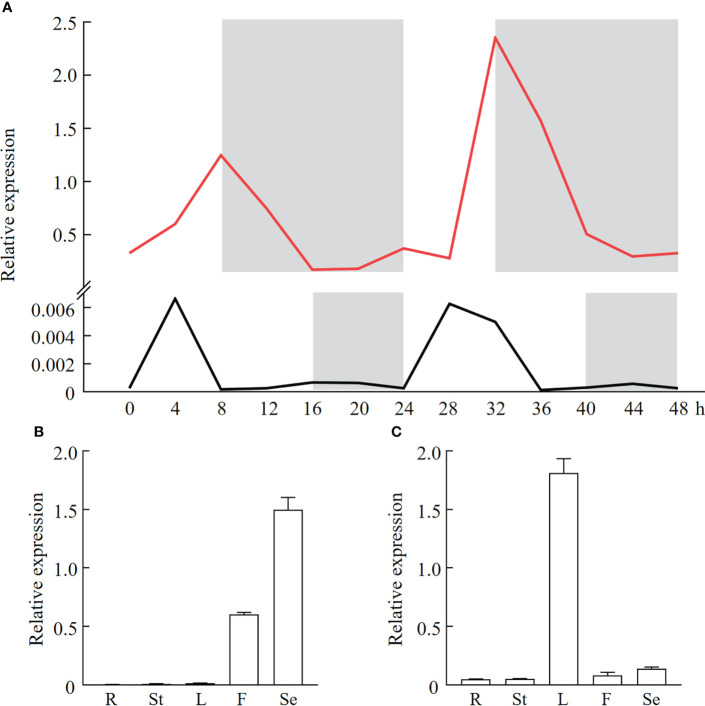
Diurnal expression pattern and tissue specificity analysis of *PfFT1* in *P. frutescens*. **(A)** Diurnal expression pattern of *PfFT1* in leaves under the SD (red line) or LD (black line) photoperiod. The dark duration was shaded with gray. Relative expression level of *PfFT1* in different tissues under the SD **(B)** or LD **(C)** photoperiod. R, roots; St, stems; L, leaves; F, flowers; Se, seeds. The roots, stems, leaves, flowers at flowering stage (56/141 DAS) were collected, and mature seeds were collected at 86/171 DAS under SD and LD condition, respectively.

To understand effects of photoperiod on *PfFT1* expression, its expression within 48 h under LD or SD conditions was analyzed by RT-qPCR (**Figure 6A**). The result revealed that *PfFT1* have specific rhythmic expression patterns under different photoperiod conditions. Under the SD conditions, its expression level was increased with the increase of light duration within 0-8 h, then gradually decreased under the dark condition (8-24 h). Within 24-48h, its expression level peaked after 8 h of light (32 h), but it was higher than that within 0-24 h. Under the LD condition, the expression level reached a peak after 4 h light exposure (significantly lower than the peak value under the SD condition), and then gradually decreased to a very low level. In conclusion, the expression level of *PfFT1* under the LD condition was extremely low, which strongly supported that the *Perilla* is a SD plant.

To further explore the relationship between the *PfFT1* expression and flowering, its tissue expression pattern under different photoperiods was analyzed (**Figures 6B, C**). Under the LD condition, the highest expression was detected in seeds, then followed by flowers, while it was extremely low in roots, stems and leaves. Under the SD condition, highest expression was found in leaves, followed by flowers and seeds. But the expression was very low in roots and stems. These results suggested that *PfFT1* could promote flowering under SD conditions, whereas it might also regulate seed development under LD conditions.

### Promotion effect of *PfFT1* gene on flowering in *Arabidopsis*

**Figure 7:**
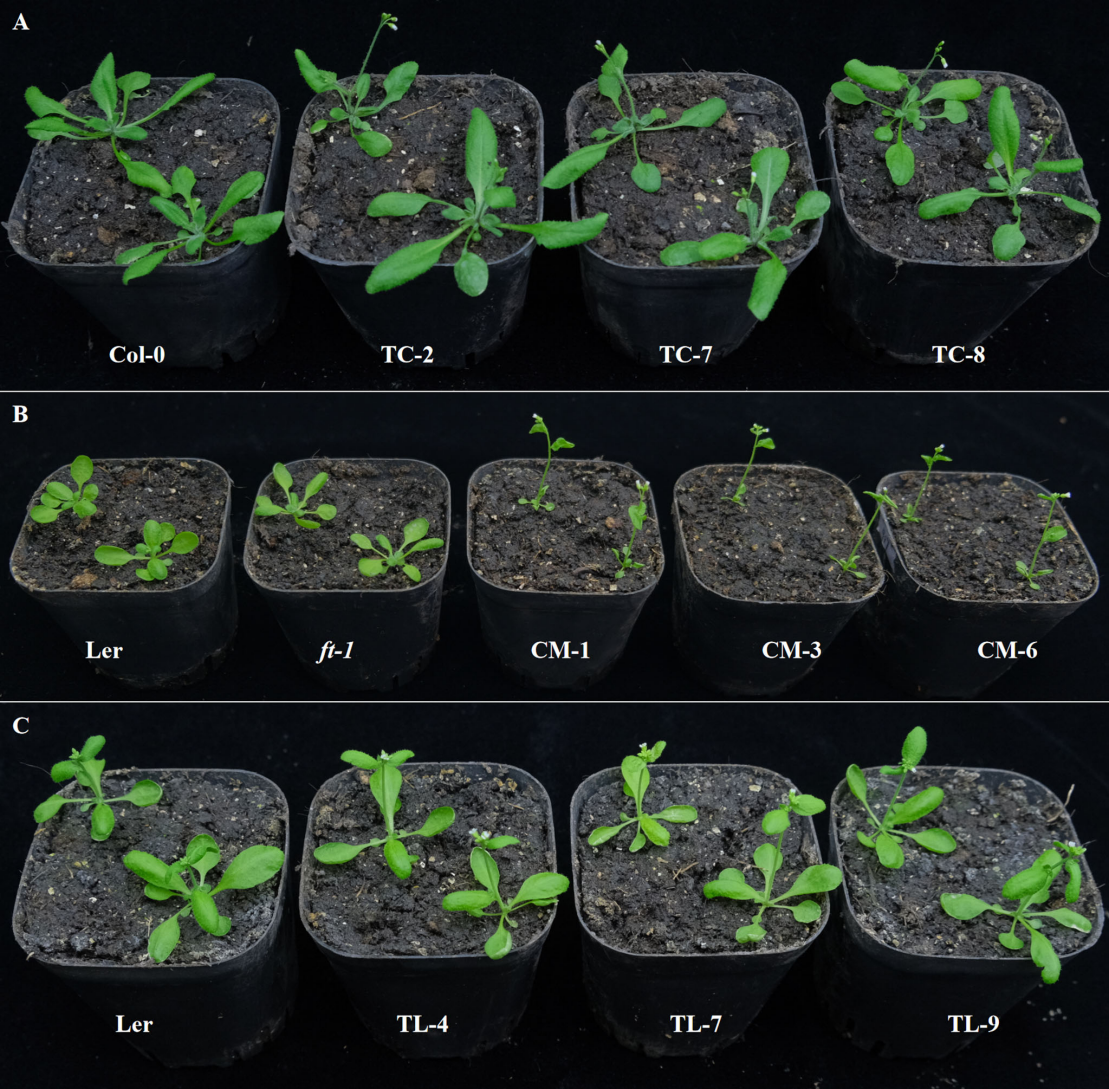
Flowering traits of transgenic *Arabidopsis* lines. **(A)** From left to right are Col-0, TC-2, TC-7, and TC-8 (29^th^ DAS). **(B)** From left to right are Ler, *ft-1*, CM-1, CM-3, and CM-6 (20^th^ DAS). **(C)** From left to right are Ler, TL-4, TL-7, and TL-9 (22^th^ DAS).

To study the regulatory role of *PfFT1* on flowering, *PfFT1* gene driven by CaMV 35S promoter was transferred into *Arabidopsis* Col-0, Ler, and *ft-1*, respectively (**Figures S3A, B**). After hygromycin screening, GUS staining and PCR identification, a total of 18 TC, 10 CM and 15 TL transgenic lines were obtained (**Figures S3C–F**). Subsequently, three T_3_ plants of each transgenic line were selected based on the expression level of *PfFT1* for further analysis. Compared with the controls, all transgenic plants exhibited varying degrees of early flowering (**Figures 7A–C**). Compared with the wild-type Col-0, the bolting and flowering time of TC lines were 5-8 day earlier with 2-3 less rosette leaves at flowering (**Table 1**). Overexpression of *PfFT1* not only promoted early flowering of Col-0, but also rescued the flowering time defect of *ft-1* mutant. All CM lines started bolting at day 15-16, the flowering time was 30 d earlier than that of *ft-1* line, and the number of rosette leaves at flowering was only 4-5. However, the *ft-1* mutant bloomed around day 50 with 18 rosette leaves (**Table 2**). These results indicated that the overexpression of *PfFT1* could complemented the delayed flowering in *ft-1* mutant. In addition, it was found that the bolting and flowering time of TL lines were 3-5 d earlier than that of Ler type (**Table 2**).

**Table 1:** Bolting traits of Col-0 and transgenic Col-0 (TC) *Arabidopsis*.

Type	Bolting time (DAS)	Flowering time (DAS)	Number of rosette leaves
Col-0	27.36 ± 1.57^a^	36.55 ± 1.75^a^	12.91 ± 1.36^a^
TC-2	20.36 ± 1.50^b^	28.91 ± 1.14^b^	9.91 ± 1.05^b^
TC-7	20.64 ± 1.27^b^	29.27 ± 1.27^b^	10.18 ± 1.47^b^
TC-8	21.82 ± 1.47^b^	29.82 ± 1.17^b^	10.18 ± 1.09^b^

Values represent means ± standard deviation (SD). Different letters in the same column indicate significant differences (P<0.05).

**Table 2:** Bolting traits of Ler, *ft-1*, complemented mutant (CM), and transgenic Ler (TL) *Arabidopsis*.

Type	Bolting time (DAS)	Flowering time (DAS)	Number of rosette leaves
Ler	20.20 ± 1.01^b^	25.27 ± 0.91^b^	9.37 ± 1.03^b^
*ft-1*	39.64 ± 1.29^a^	50.27 ± 2.01^a^	17.91 ± 1.59^a^
CM-1	15.46 ± 1.21^c^	19.18 ± 1.17^c^	4.55 ± 0.69^d^
CM-3	16.09 ± 1.58^c^	19.73 ± 1.27^c^	4.36 ± 0.67^d^
CM-6	15.73 ± 1.27^c^	19.64 ± 1.21^c^	4.46 ± 0.69^d^
TL-4	16.27 ± 1.10^c^	21.27 ± 1.01^c^	7.27 ± 1.1^c^
TL-7	15.36 ± 1.36^c^	20.00 ± 1.00^c^	6.46 ± 1.04^c^
TL-9	16.64 ± 1.21^c^	21.81 ± 1.66^c^	7.55 ± 1.21^c^

Values represent means ± standard deviation (SD). Different letters in the same column indicate significant differences (P<0.05).

### Expression analysis of flowering-related endogenous genes in *Arabidopsis*

RT-qPCR analysis showed that *PfTF1* was expressed in all TC, CM and TL lines (**Figures S4A–C**), but was not detected in wild-type Col-0, and Ler type. Expression of *AtFT* and *PfFT1* were not detected in *ft-1* mutant (**Figures S4B, E**).

**Figure 8:**
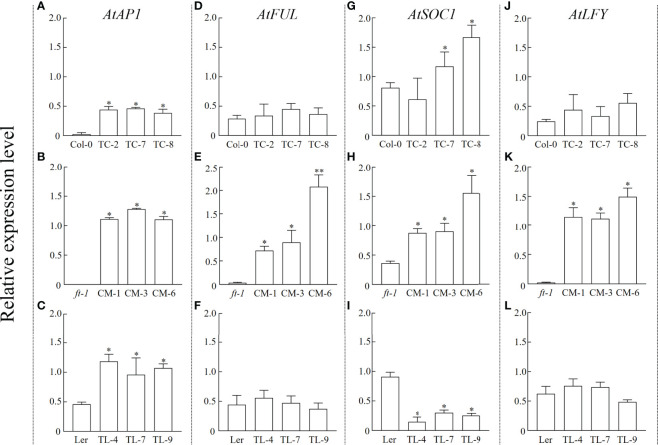
Relative expression level of endogenous flowering related genes in *Arabidopsis*. Expression levels of AtAP1, AtFUL, AtSOC1, and AtLFY in Col-0 and TC lines **(A, D, G, J)**, ft-1 and CM lines **(B, E, H, K),** Ler and TL lines **(C, F, I, L),** respectively * means p < 0.05.

To verify whether the introduction of *PfFT1* could regulate the expression of endogenous flowering-related genes in *Arabidopsis*, the expression level of *AtAP1*, *AtFUL*, *AtSOC1* and *AtLFY* were analyzed (**Figures 8A–L**). In TC lines, high expression of *AtSOC1* and *AtAP1* was observed (**Figures 8A, G**). Compared with the *ft-1* mutant, *AtAP1*, *AtFUL*, *AtSOC1* and *AtLFY* were significantly up-regulated in CM lines (**Figures 8B, E, H, K**). Compared with the non-transgenic *ft-1* line, the flowering time of transgenic lines was significantly earlier. It was also earlier than the average flowering time of Ler (25.27 ± 0.91) (**Table 2**). Compared with the non-transgenic Ler, *AtAP1* was highly expressed in OL lines, but *AtSOC1* was less expressed (**Figures 8C, I**).

## Discussion

In plants, the PEBP family is a class of proteins with the conserved PEBP domain, and involved in inflorescence structure and seed development, and germination (Yu et al., 2019; Schmidt et al., 2020; Zhang et al., 2020). However, their functions in *Perilla* have not been studied. Therefore, studies on the PfPEBP family will be helpful for the genetic breeding and improvement of yield traits of *P. frutescens*.

In this study, a total of 10 *PfPEBP* genes were identified in the *P. frutescens* genome, and all consist of 4 exons and 3 introns. Phylogenetic analysis showed that PfPEBP were clustered into three subfamilies: FT-like, TFL1-like and MFT-like. Members in a same subfamily have the conserved motif DPDxP and GxHR, indicating that they are evolutionarily conserved. Previous studies found that the substitution of key amino acids in the PEBP domain (Tyr^85^→His^88^; Gln^140^→Asp^144^) resulted in the opposite roles of FT and TFL1 in regulating plant flowering (Hanzawa et al., 2005; Ahn et al., 2006). The amino acid residues encoded by the fourth exon of *FT* and *TFL1* determine their functional specificity. The corresponding part was divided into four fragments. Segment B and C (LYN motif) are crucial for FT-induced flowering (Ahn et al., 2006).

Prediction of PPI networks helps to explore potential roles of *PfPEBP*. In the photoperiod regulation pathway, the photoreceptor receives the light signal and transmits to CONSTANS (CO) through the diurnal clock. In this pathway, CO acts as a transcriptional activator to induce plant flowering, while FLC acts as a flowering inhibitor through the vernalization pathway (Luo et al., 2019). In addition, CO and FLC also regulate the expression of downstream genes *FT*, *SOC1* (also known as *AGL20*), and *LFY*. A previous study showed that the transcription factor SHORT VEGETATIVE PHASE (SVP) binds to the CArG motif in the promoter of *FT* gene and negatively regulates its expression, thereby regulating plant flowering (Lee et al., 2007). During the vegetative growth stage, the TFL-FD complex can inhibit the expression of *LFY* gene in the meristem through specifically binding to the G-box motif in the second exon, thereby maintaining the vegetative properties of the meristem (Yamaguchi, 2021). *PfFT1* could promote flowering probably by regulating the expression of downstream flowering-related genes *AP1*, *LFY*, *FUL* and *SOC1* (**Figure 8**).

In the SD plant *Perilla* and rice, the promoter regions of *PfPEBP* genes contain specific light-response elements, such as ACE, AT1-motif, Box-4. Therefore, we speculated that they might be involved in regulating flowering. Studies have shown that the flower formation signal integrated by the photoperiod can induce the flower formation by activating *FT* expression. In rice, the highest expression of *Hd3a* was detected in leaves under the SD condition (Izawa et al., 2002; Tamaki et al., 2007). In the LD plant *Arabidopsis*, its highest expression level was also found in leaves under the LD condition (Wigge et al., 2005). In this study, the same expression pattern of *PfFT1* was detected in leaves with a diurnal rhythm under the SD condition. It was also showed that the expression level of *FT* genes was closely related to seasonal flowering of soybean (Glycine max), poplar (*Populus deltoides*) and loquat (*Eriobotrya japonica*) (Hsu et al., 2006; Reig et al., 2017; Lee et al., 2021). Our results revealed that the expression of *PfFT1* in *P. frutescens* had obvious diurnal rhythm under the SD or LD condition. A previous study has shown that PfFT1, a key protein for flowering induction, is synthesized in leaves and transported to the meristem of the shoot apex to induce flower formation (Kaneko-Suzuki et al., 2018). Under the SD condition, the high expression of *PfFT1* in leaves is expected as *Perilla* is a SD plant.

Our results indicated that the expression of *PfFT1* could promote flowering. The flowering time of the transgenic TC lines were 6-8 day earlier than that of the non-transgenic plants Col-0. Its overexpression could rescue the late flowering phenotype of *ft-1* mutant, and the flowering time was 5-6 day earlier than that of Ler type. Similarly, its overexpression in Ler type resulted in 2-3 day earlier flowering. Studies have shown that overexpression of *FT* gene could promote the development of floral organs. In cassava (*Manihot esculenta*), transgenic overexpression of the endogenous *MeFT1* resulted in early flowering by recruiting downstream floral meristem identity genes (*MeAP1*, *MeLFY* and *MeSOC1*) in shoot apical tissues (Odipio et al., 2020). The expression of *LsFT* was involved in the bolting of different lettuce (*Lactuca sativa*) varieties. Knockdown of *LsFT* by RNA interference significantly delayed bolting in lettuce (Chen et al., 2018). In addition, overexpression of *LsFT* in Arabidopsis could rescue the late-flowering phenotype of *ft-2* mutant (Chen et al., 2018). In *Medicago truncatula*, *MtFTa1* and *MtFDa* were considered as key flowering regulators, *MtFDa* was essential for floral transition and secondary inflorescence development (Cheng et al., 2021). In this study, early flowering of transgenic *Arabidopsis* was positively correlated with the expression levels of floral meristem recognition genes *AtAP1*, *AtLFY* and *AtFUL*, which was consistent with the findings in *M. esculenta* (Odipio et al., 2020). Our findings lay a foundation for further researches on *PfPEBP* genes in regulating flowering of *P. frutescens*.

## Conclusion

Ten *PfPEBP* genes were identified in the rigorous SD plant *Perilla*, and divided into three subfamilies (FT-like, TFL1-like, and MFT-like) based on their phylogenetic relationships and gene structure characteristics. *Cis*-regulatory element analysis revealed that the promoter region of each *PfPEBP* genes contains many light-response elements. The expression of *PfFT1* is regulated by the photoperiod with tissue specificity and diurnal rhythm. The transgenic *Arabidopsis* lines overexpressing *PfFT1* gene exhibit an early flowering phenotype. We speculated that the molecular mechanism of flowering promotion by *PfFT1* is to activate expressions of regulatory genes *AtAP1*, *AtFUL*, *AtSOC1* and *AtLFY*. In conclusion, results laid a foundation for elucidating the molecular mechanism of *PfFT1* gene regulating flowering and genetic traits improvement of *P. frutescens*.

## Data availability statement

The original contributions presented in the study are included in the article/**Supplementary Material**. Further inquiries can be directed to the corresponding author.

## Author contributions

HX, YH, and TZ designed the experiments and wrote the manuscript. HX, XG, and DL executed the experiments and prepared the Figures. HX, XG, GL, and JL analyzed the data. All authors contributed to the article and approved the submitted version.

## Funding

This research was funded by the National Natural Science Foundation of China (Project No. 31171588), Science and Technology Research Program of Chongqing Municipal Education Commission (Project No. KJZD-K202200508). This work was also supported by the Graduate Research Innovation Project of Chongqing Normal University (Project No. YKC21033).

## Conflict of interest

The authors declare that the research was conducted in the absence of any commercial or financial relationships that could be construed as a potential conflict of interest.

## Publisher’s note

All claims expressed in this article are solely those of the authors and do not necessarily represent those of their affiliated organizations, or those of the publisher, the editors and the reviewers. Any product that may be evaluated in this article, or claim that may be made by its manufacturer, is not guaranteed or endorsed by the publisher.

## Supplementary material

Supplementary Figure 1 Chromosome distribution of *PfPEBP* genes. The chromosomal locations of *PfPEBP* genes were mapped based on genome annotation. The scale is in mage bases (Mb).

Supplementary Figure 2 Multiple sequence alignment for PPI network prediction. Multiple sequence alignment of PfFTs and AtFT **(A)**, PfTFL1s and AtTFL1 **(B)** was performed by Clustal Omega.

Supplementary Figure 3 Cloning of *PfFT1* genes and identification of transgenic Arabidopsis. **(A)** Agrose gel electrophoresis of *PfFT1* amplified from *Perilla* cDNA. M, DNA Marker (from bottom to top) were 100, 250, 500, 750, 1000, and 2000 bp, sequentially; 1, Fragment amplified with PfFT1-F and PfFT1-R primers. **(B)** Detailed information of vector pCAMBIA1303-*PfFT1*. **(C)** Transgenic lines screened on the medium with hygromycin B. **(D)** Identification of transgenic lines by PCR amplification of target *PfFT1* gene. M: DNA Marker; 1-3, line OE-2,OE-7,OE-8, 4-6, line CM-1,CM-3,CM-6, 7-9, line OL-4,OL-7, OL-9, 10-13, line Col, ft-1, Ler, and control (ddH2O). **(E)** Identification of transgenic lines by GUS staining.

Supplementary Figure 4 Expression analysis of *PfFT1* and endogenious gene *AtFT* in transgenic Arabidopsis. Expression level of PfFT1, AtFT1 in Col-0 and TC line **(A, D),** ft-1 and CM line **(B, E),** Ler and TL line **(C, F)**, respectively.
