# Supplementary material for: Genome-wide characterization of PEBP gene family in Perilla frutescens and PfFT1 promotes flowering time in Arabidopsis thaliana
Source: Front Plant Sci. 2022 Nov 18;13:1026696. doi: 10.3389/fpls.2022.1026696 (PMC9716100; doi:10.3389/fpls.2022.1026696)
Supplement: Supplementary Figure 1 — Chromosome distribution of PfPEBP genes. The chromosomal locations of PfPEBP genes were mapped based on genome annotation. The scale is in mage bases (Mb). [file DataSheet_1.docx]

Supplementary Material

# Supplementary Data

The Supplementary Material for this article can be found in additional files (Supplementary Tables S1 to S6, and Figures S1 to S4).

Supplementary Table S1 List of primer sequences in this study.

Supplementary Table S2 Complete list of *PEBP* family genes identified in the *P. frutescens* genome.

Supplementary Table S3 Protein accession numbers of the sequences used in N-J tree.

Supplementary Table S4 List of *cis*-regulatory elements in *PfPEBP* family genes.

Supplementary Table S5 List of *cis*-regulatory elements in *PfFT1*, *OsFTL1*–*3*, *AtFT*.

Supplementary Table S6 Percent Identity Matrix of PfPEBPs (%).

FIGURE S1. Chromosome distribution of *PfPEBP* genes.

FIGURE S2. Multiple sequence alignment for PPI network prediction.

FIGURE S3. Cloning of *PfFT1* genes and identification of transgenic *Arabidopsis*.

FIGURE S4 Expression analysis of *PfFT1* and endogenious gene *AtFT* in transgenic *Arabidopsis*.

# Supplementary Figures and Tables


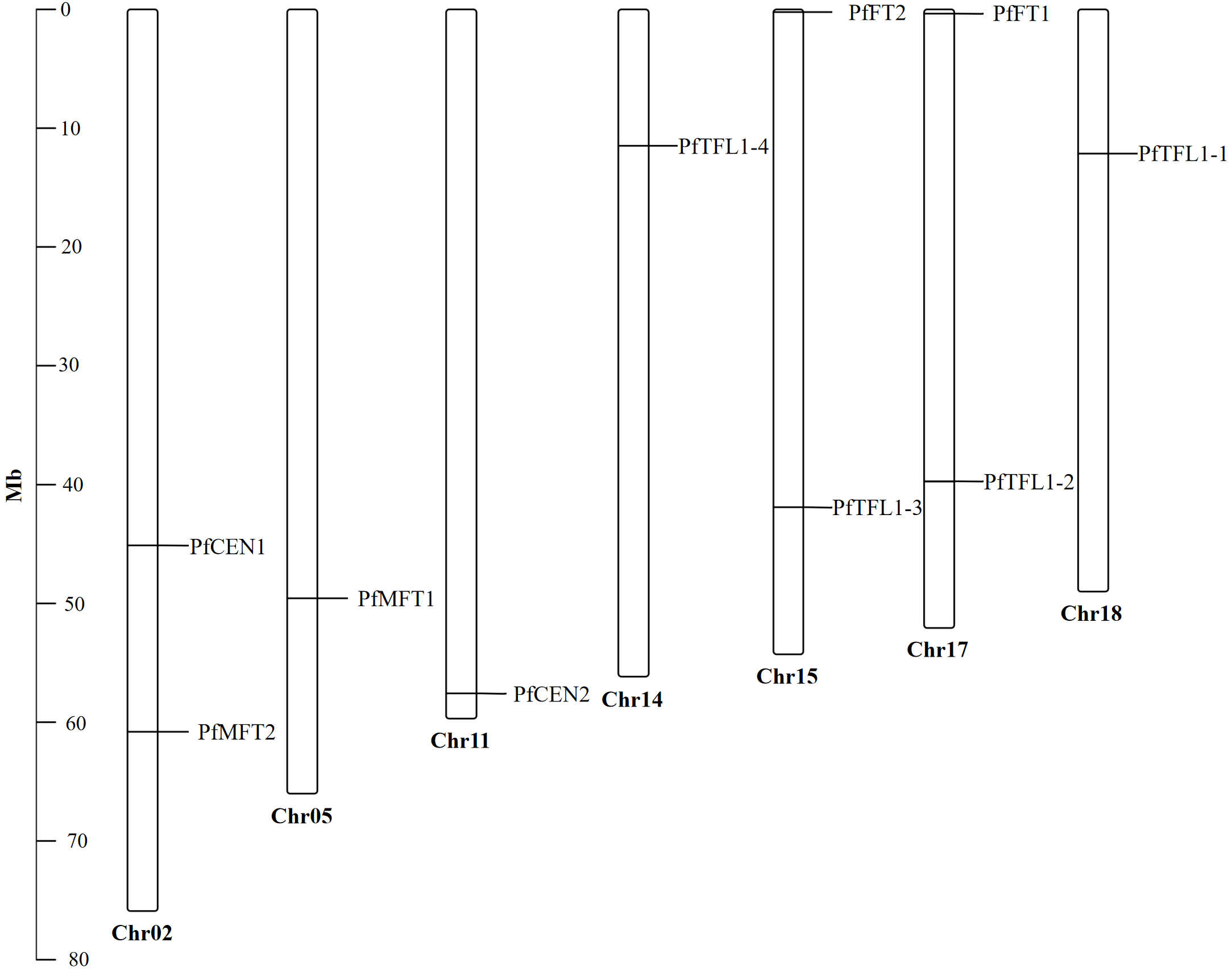


**FIGURE S1. Chromosome distribution of *PfPEBP* genes.** The chromosomal locations of *PfPEBP* genes were mapped according to the *P. frutescens* genome sequence. The scale is in mage bases (Mb).


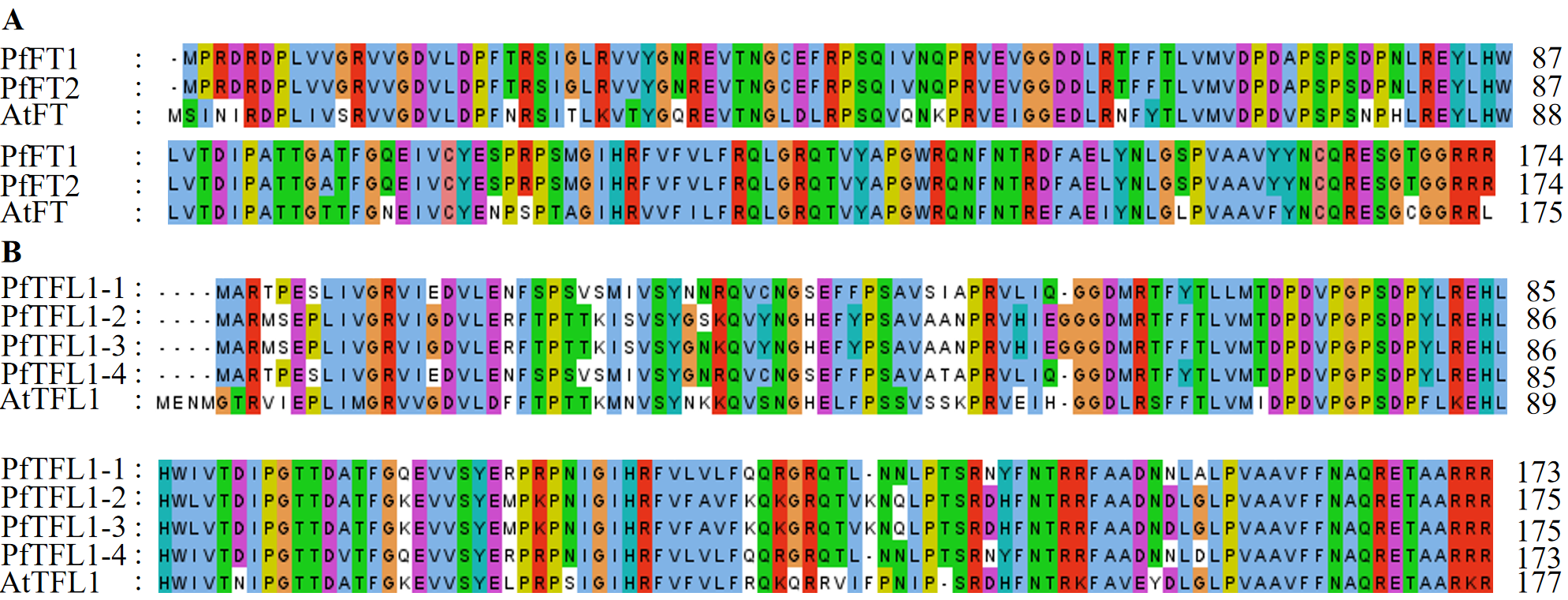


**FIGURE S2. Multiple sequence alignment for PPI network prediction.** Multiple sequence alignment of PfFTs and AtFT **(A)**, PfTFL1s and AtTFL1 **(B)** was performed by Clustal Omega.

**
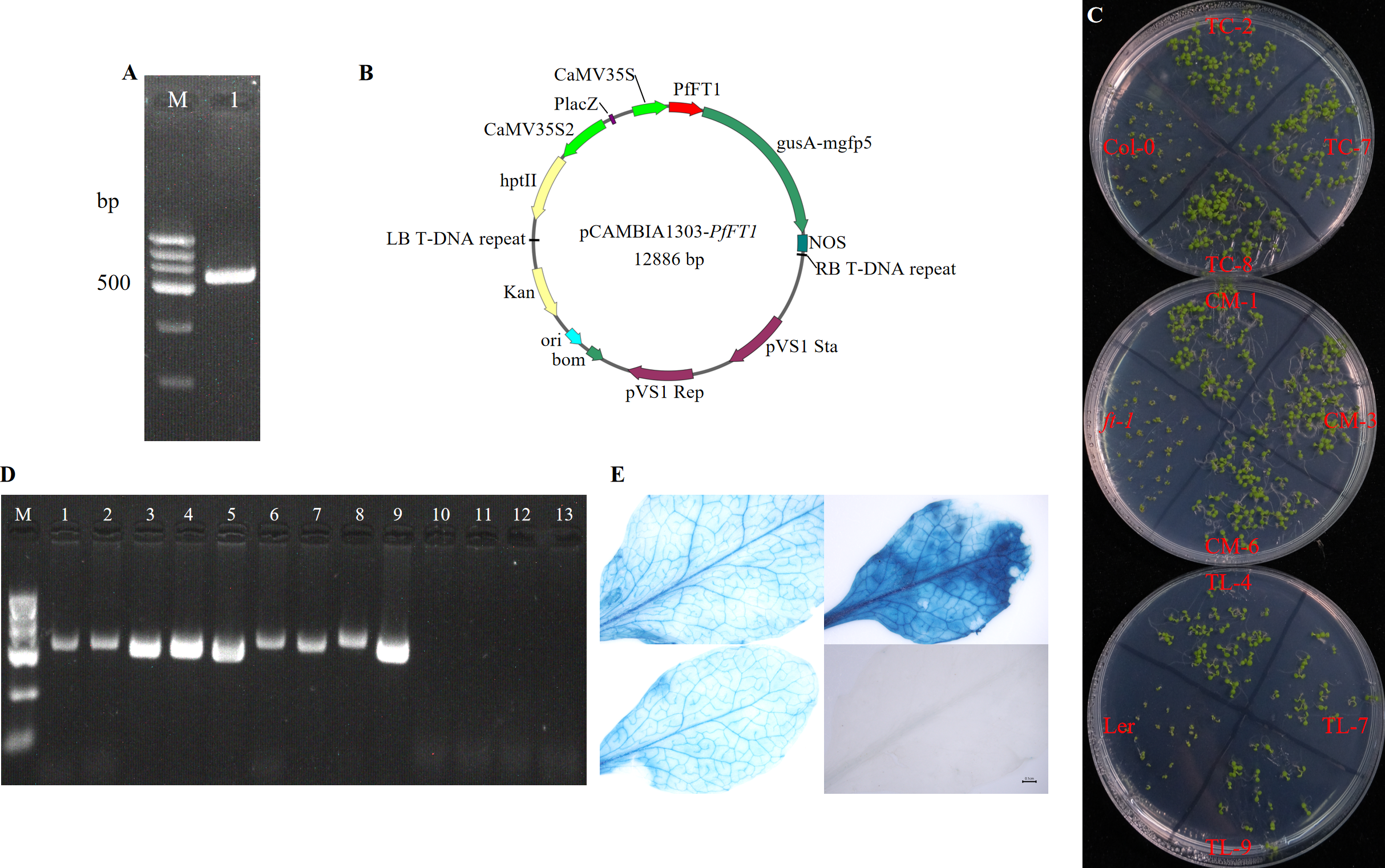
**

**FIGURE S3. Cloning of PfFT1 genes and identification of transgenic *Arabidopsis*.** **(A)** Agrose gel electrophoresis of PCR product of *PfFT1* amplified from *Perilla* cDNA. 1: Fragment amplified with PfFT1-F and PfFT1-R primers; M: DNA Marker (100~2000 bp, from bottom to top were 100, 250, 500, 750, 1000, and 2000 bp, sequentially). **(B)** Detailed information of vector pCAMBIA1303-*PfFT1*. **(C)** Transgenic line screening on the medium with Hygromycin B. **(D)** Identification of transgenic line by PCR amplification of target *PfFT1* gene. M: DNA Marker (100~2000 bp), 1-3 means OE-2,OE-7,OE-8 lines, 4-6 means CM-1,CM-3,CM-6 lines, 7-9 means OL-4,OL-7, OL-9 lines, 10-13 means Col, *ft-1*, Ler, and ddH_2_O, respectively. **(E)** Identification of transgenic line by GUS staining.

**
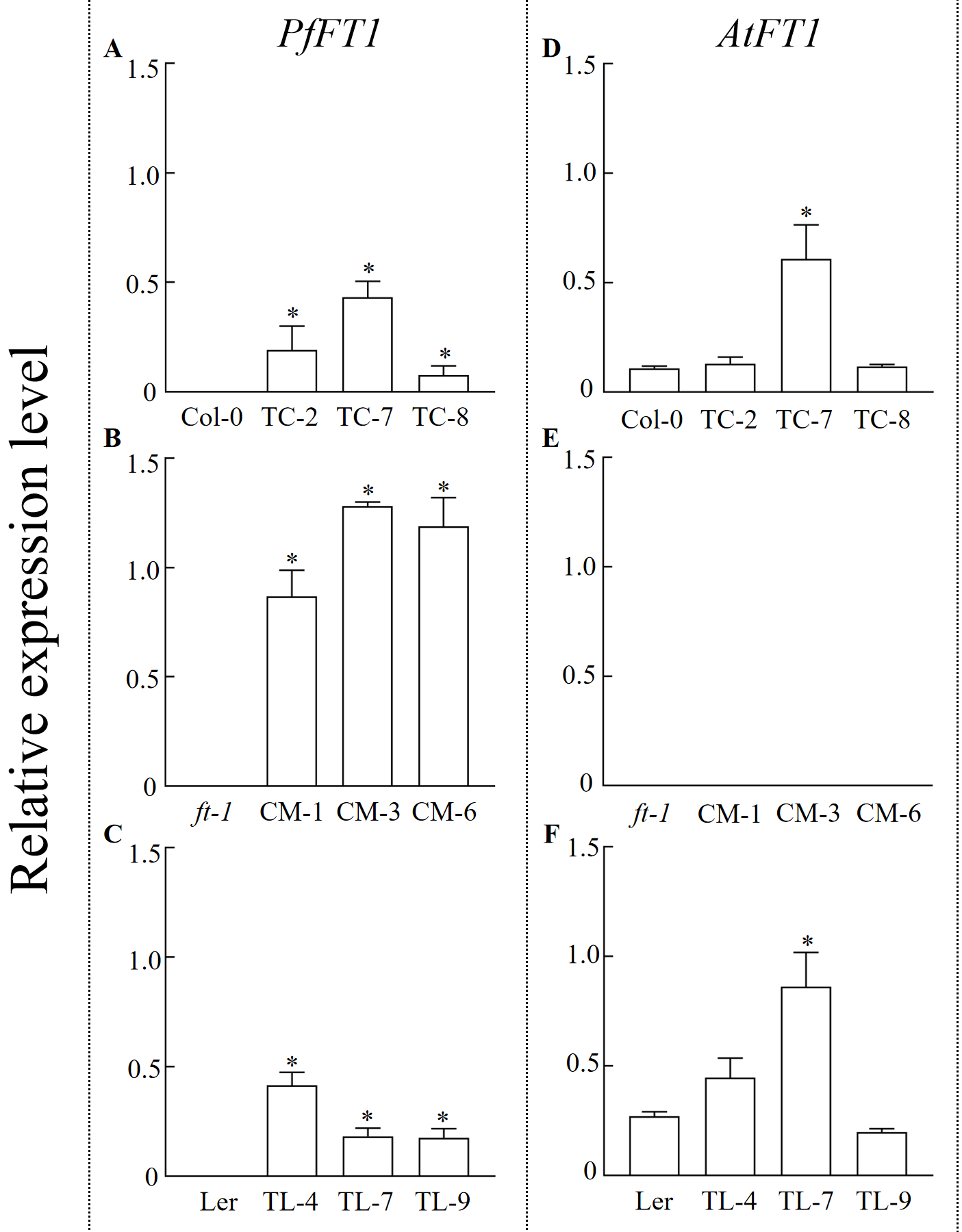
**

**FIGURE S4 Expression analysis of *PfFT1* and endogenious gene *AtFT* in transgenic *Arabidopsi*s.** Expression level of *PfFT1* in Col-0 and TC line **(A)**, *ft-1* and CM line **(B)**, Ler and TL line **(C)**. Expression level of *AtFT1* in Col-0 and TC line **(D)**, *ft-1* and CM line **(E)**, Ler and TL line **(F)**.
